# Supplementary material for: DFT Studies of Dimethylaminophenyl-Substituted Phthalocyanine and Its Silver Complexes
Source: Molecules. 2024 Mar 18;29(6):1344. doi: 10.3390/molecules29061344 (PMC10974634; doi:10.3390/molecules29061344)
Supplement: Supplementary file 1 [file molecules-29-01344-s001.zip › molecules-2879151-supplementary.pdf]

DFT studies of dimethylaminophenyl-substituted phthalocyanine and of its silver complexes

Martin Breza

Department of Physical Chemistry, Slovak Technical University, Radlinskeho 9, SK-81237 Bratislava, Slovakia

Supplementary information

Figure S1. UV-vis spectra of neutral dmaphPcAg (full line) and dmaphPcH_2 (dashed line) in $CHCl_3$.

Figure S2. Time dependence of absorption spectra during photolysis of $[dmaphPcAg]^0$ under LED@385 nm irradiation.

Figure S3. DFT optimized structure of $^2[dmaphPcH]^0$ in $CHCl_3$.

Figure S4. DFT optimized structure of $^2[dmaphPc]^-$ in $CHCl_3$.

Figure S5. DFT calculated spin density of $^4[dmaphPcAg]^0$ in $CHCl_3$.

Figure S6. DFT calculated spin density of $^3[dmaphPcAg]^-$ in $CHCl_3$.

Figure S7. DFT calculated spin density of $^4[dmaphPcAg]^{2-}$ in $CHCl_3$.

Figure S8. DFT calculated spin density of $^2[dmaphPcH]^0$ in $CHCl_3$

Figure S9. DFT calculated spin density of $^2[dmaphPc]^-$ in $CHCl_3$

Figure S10. TD-DFT calculated electron transitions in $^1[dmaphPcAg]^+$ in $CHCl_3$.

Figure S11. TD-DFT calculated electron transitions in $^3[dmaphPcAg]^+$ in $CHCl_3$.

Figure S12. TD-DFT calculated electron transitions in $^2[dmaphPcAg]^0$ in $CHCl_3$.

Figure S13. TD-DFT calculated electron transitions in $^4[dmaphPcAg]^0$ in $CHCl_3$.

Figure S14. TD-DFT calculated electron transitions in $^1[dmaphPcAg]^-$ in $CHCl_3$.

Figure S15. TD-DFT calculated electron transitions in $^3[dmaphPcAg]^-$ in $CHCl_3$.

Figure S16. TD-DFT calculated electron transitions in $^2[dmaphPcAg]^{2-}$ in $CHCl_3$.

Figure S17. TD-DFT calculated electron transitions in $^4[dmaphPcAg]^{2-}$ in $CHCl_3$.

Figure S18. TD-DFT calculated electron transitions in $^1[dmaphPcH_2]^0$ in $CHCl_3$.

Figure S19. TD-DFT calculated electron transitions in $^1[dmaphPcH]^-$ in $CHCl_3$.

Figure S20. TD-DFT calculated electron transitions in $^2[dmaphPcH]^0$ in $CHCl_3$.

Figure S21. TD-DFT calculated electron transitions in $^1[dmaphPc]^{2-}$ in $CHCl_3$.

Figure S22. TD-DFT calculated electron transitions in $^2[dmaphPc]^-$ in $CHCl_3$.

References

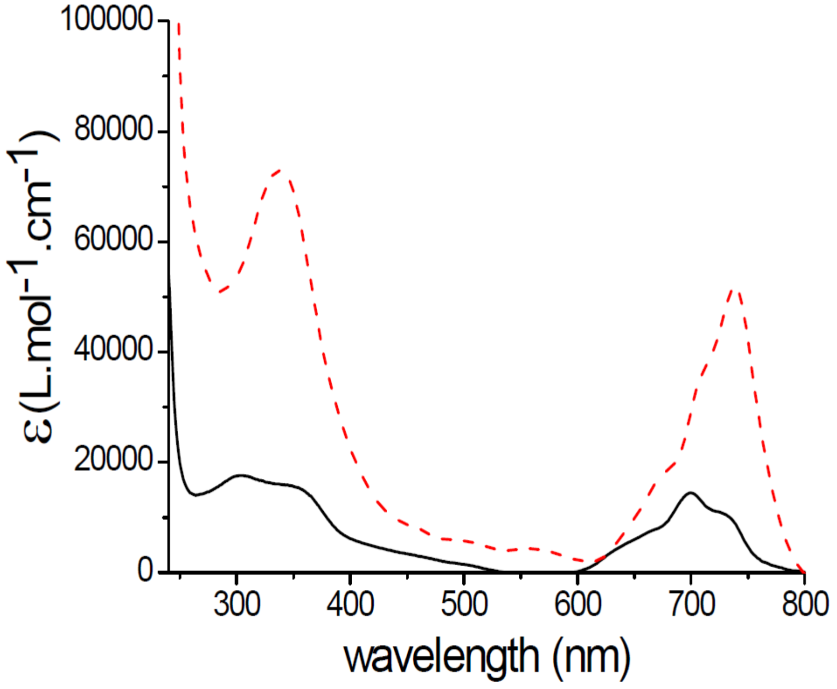

Figure S1. UV-vis spectra of neutral dmaphPcAg (full line) and dmaphPcH_2 (dashed line) in $CHCl_3$. Reproduced from Ref. [1] with permission from the Royal Society of Chemistry.

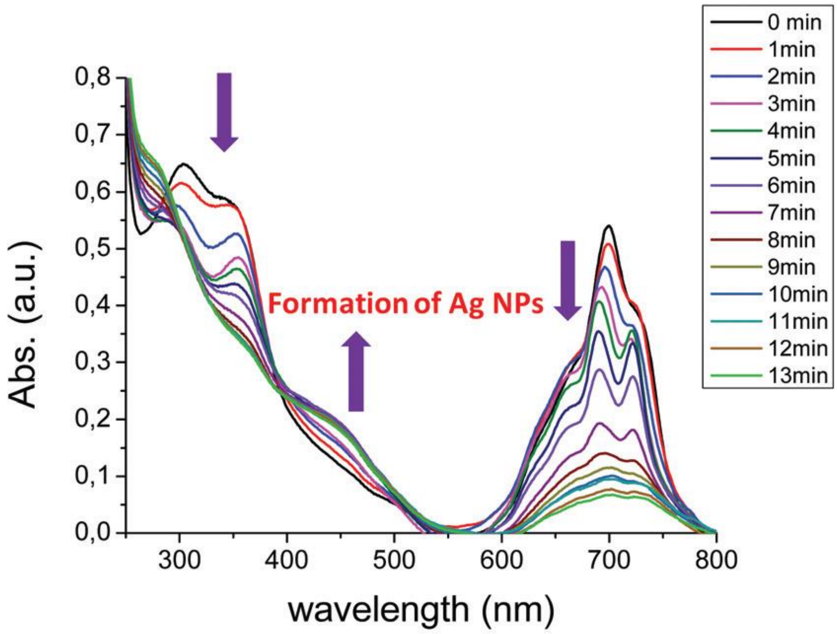

Figure S2. Time dependence of absorption spectra during photolysis of [dmaphPcAg]^0 under LED@385 nm irradiation. LED@385 nm intensity = 470 mW cm^{-2}. Concentration of [dmaphPcAg]^0 = 3.8×10^{-5} M. Solvent = $CHCl_3$. Reproduced from Ref. [1] with permission from the Royal Society of Chemistry.

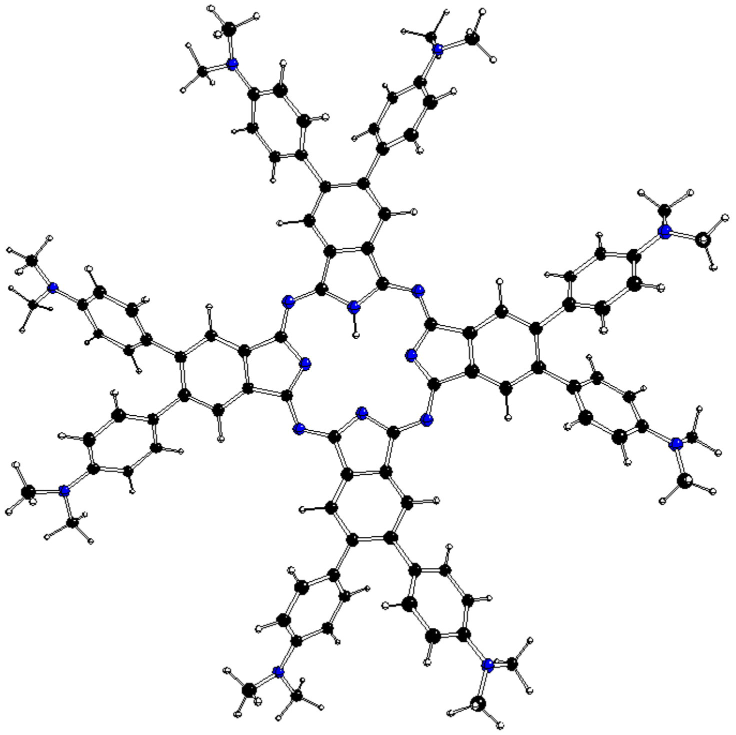

Figure S3. DFT optimized structure of $^{2}[dmaphPcH]^{0}$ in $CHCl_3$ (C – black, N – blue, H – white).

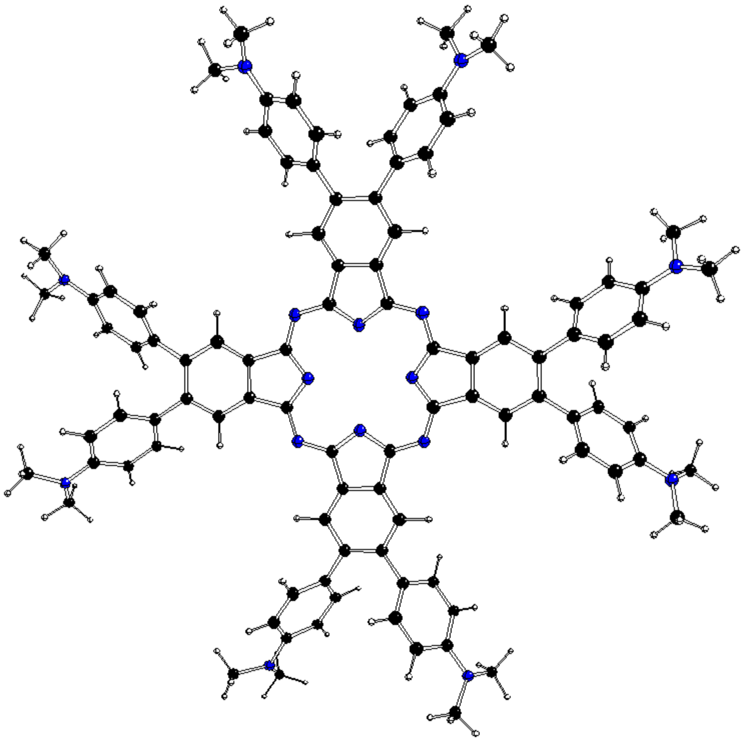

Figure S4. DFT optimized structure of $^{2}[dmaphPc]^{-}$ in $CHCl_3$ (C – black, N – blue, H – white).

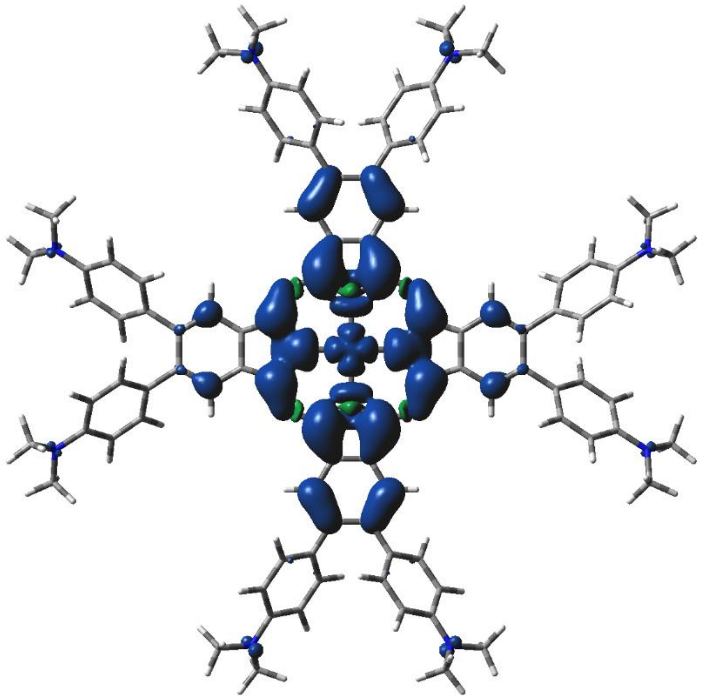

Figure S5. DFT calculated spin density of ^{4}[dmaphPcAg]^{0} in $CHCl_3$ (0.001 a.u. isosurface).

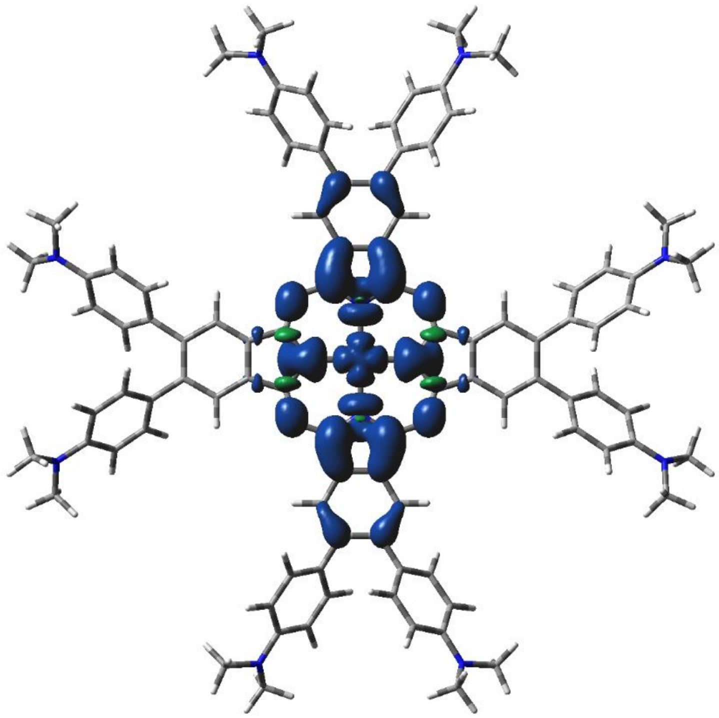

Figure S6. DFT calculated spin density of ^{3}[dmaphPcAg]$^{-}$ in $CHCl_3$ (0.001 a.u. isosurface).

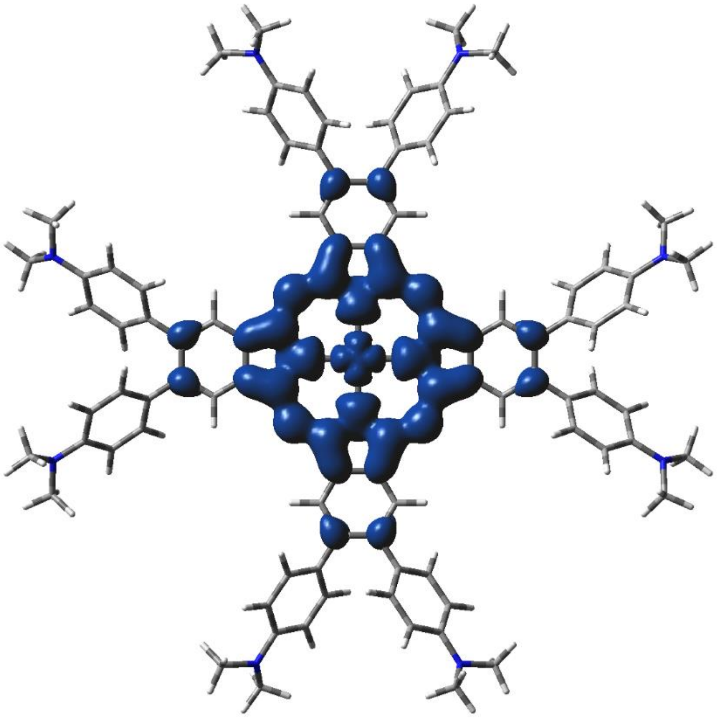

Figure S7. DFT calculated spin density of ^{4}[dmaphPcAg]$^{2-}$ in $CHCl_3$ (0.001 a.u. isosurface).

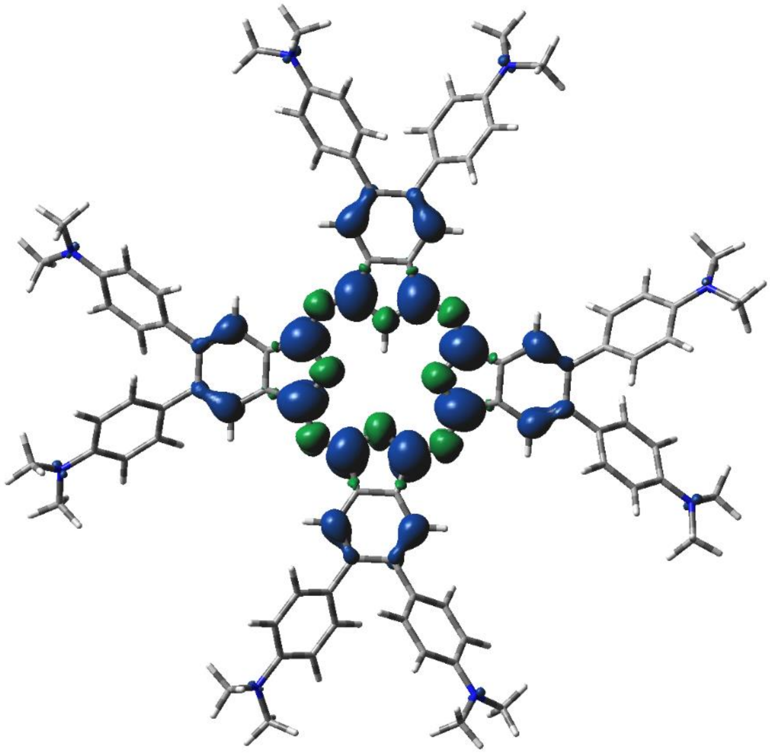

Figure S8. DFT calculated spin density of ^{2}[dmaphPcH]^{0} in $CHCl_3$ (0.001 a.u. isosurface).

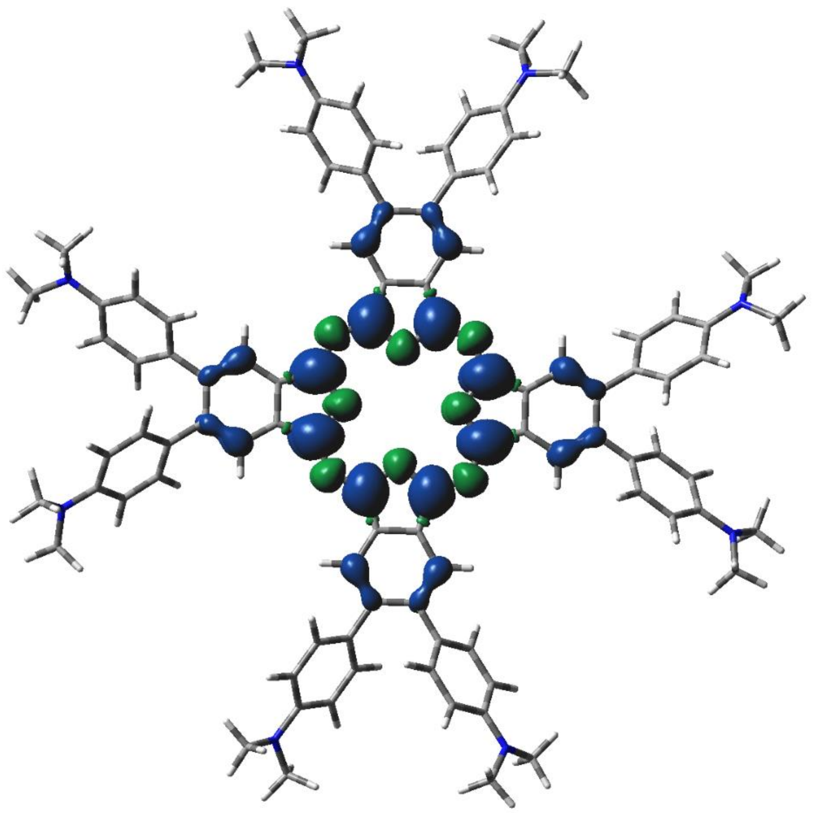

Figure S9. DFT calculated spin density of $^{2}[dmaphPc]^{-}$ in $CHCl_3$ (0.001 a.u. isosurface).

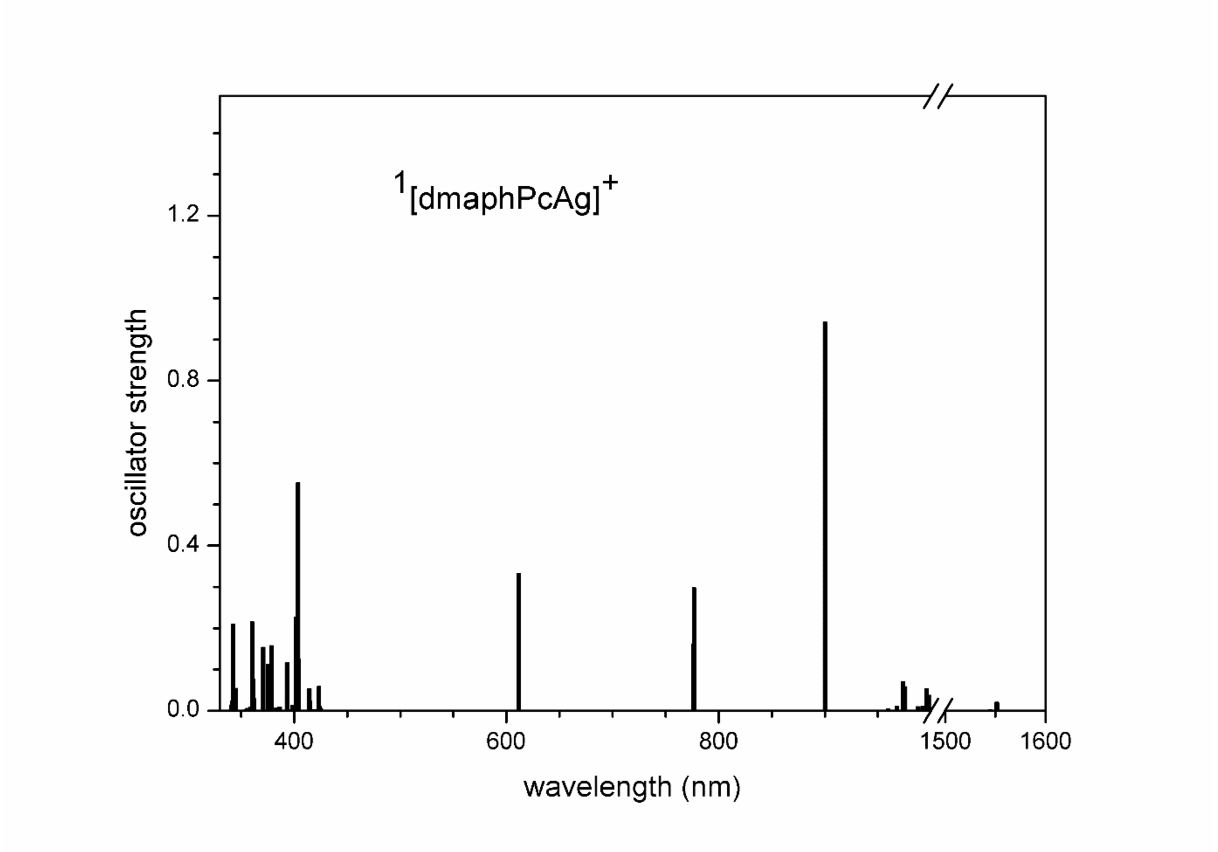

Figure S10. TD-DFT calculated electron transitions in $^{1}[dmaphPcAg]^{+}$ in $CHCl_3$.

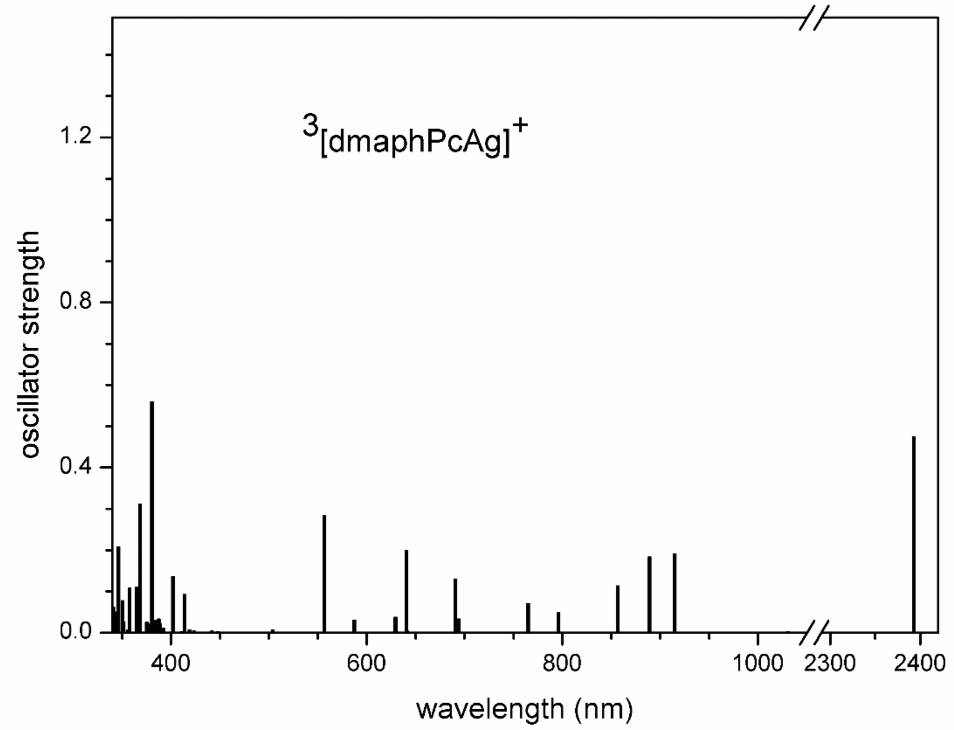

Figure S11. TD-DFT calculated electron transitions in ^{3}[dmaphPcAg]$^{+}$ in $CHCl_3$.

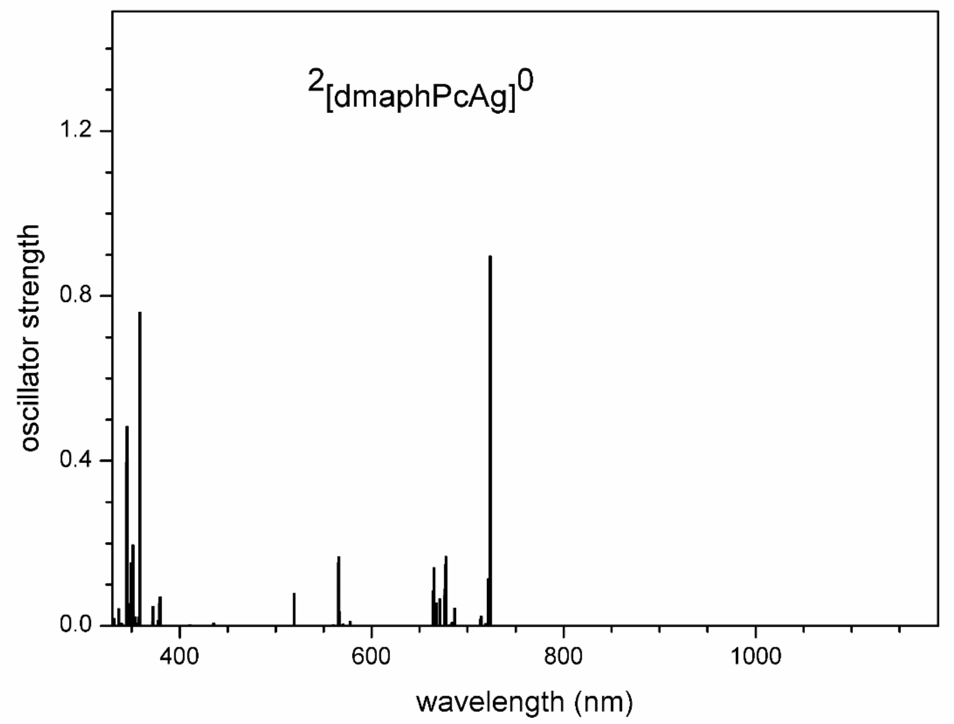

Figure S12. TD-DFT calculated electron transitions in ^{2}[dmaphPcAg]^{0} in $CHCl_3$.

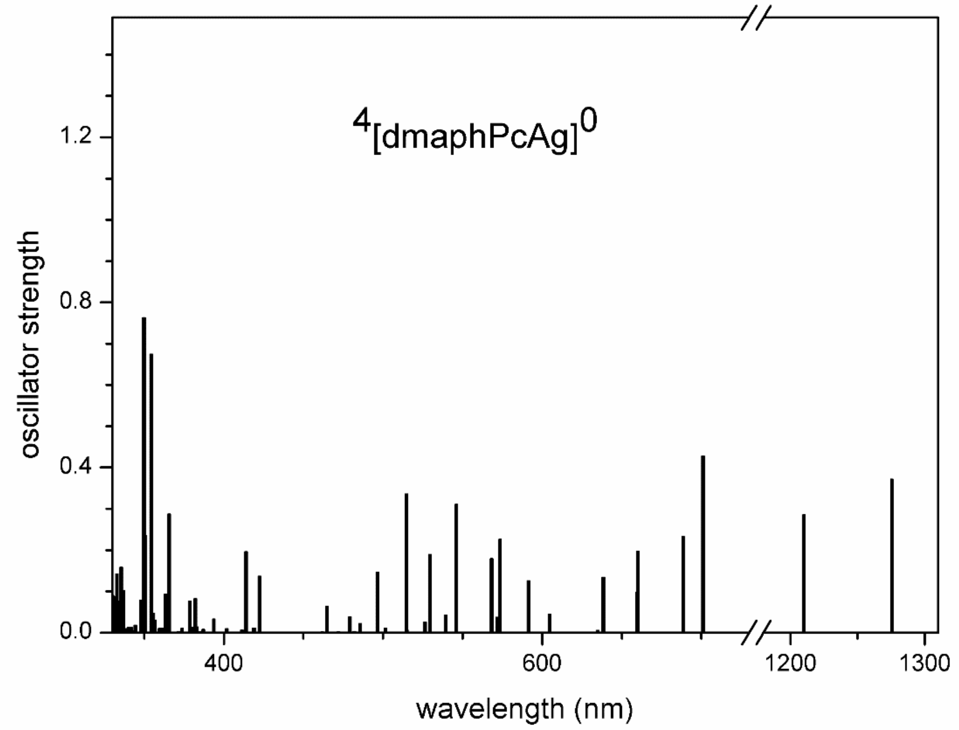

Figure S13. TD-DFT calculated electron transitions in ^{4}[dmaphPcAg]^{0} in $CHCl_3$.

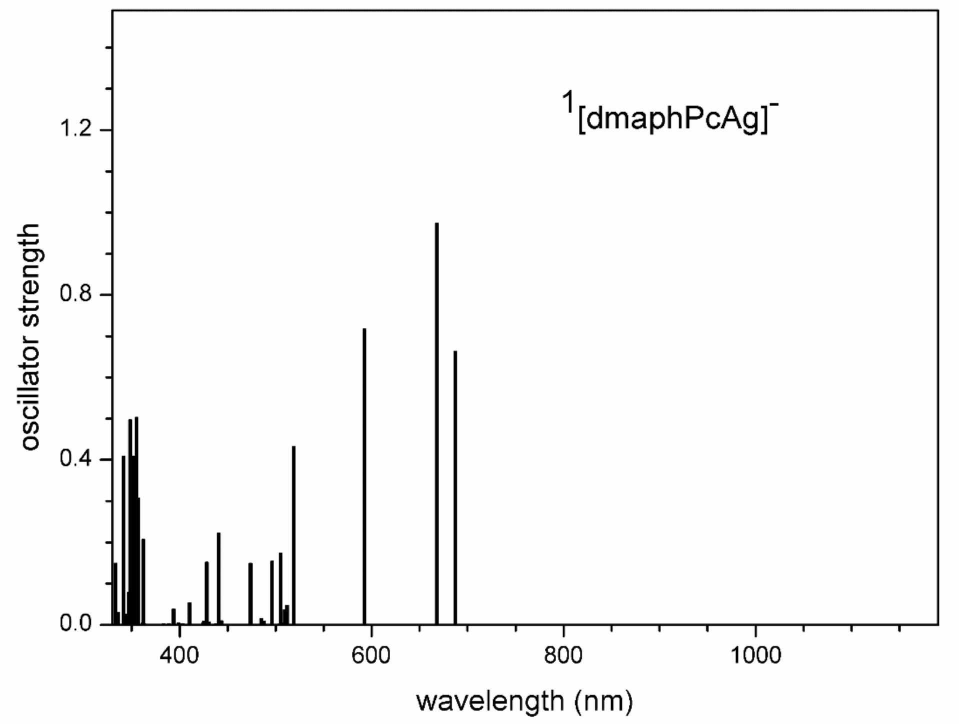

Figure S14. TD-DFT calculated electron transitions in ^{1}[dmaphPcAg]$^{-}$ in $CHCl_3$.

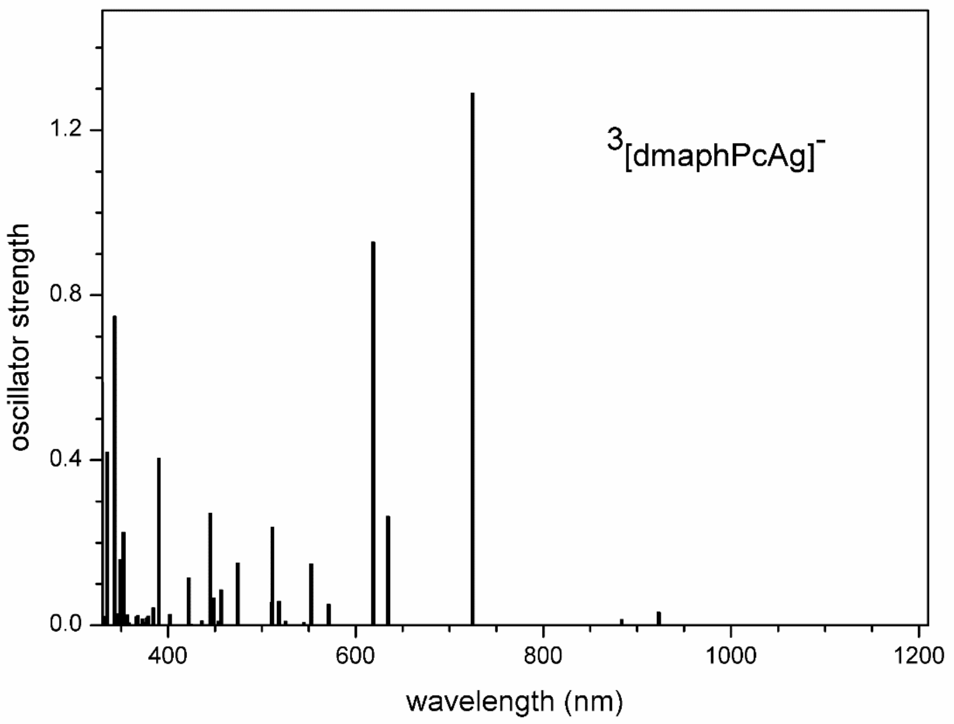

Figure S15. TD-DFT calculated electron transitions in ^{3}[dmaphPcAg]$^-$ in $CHCl_3$.

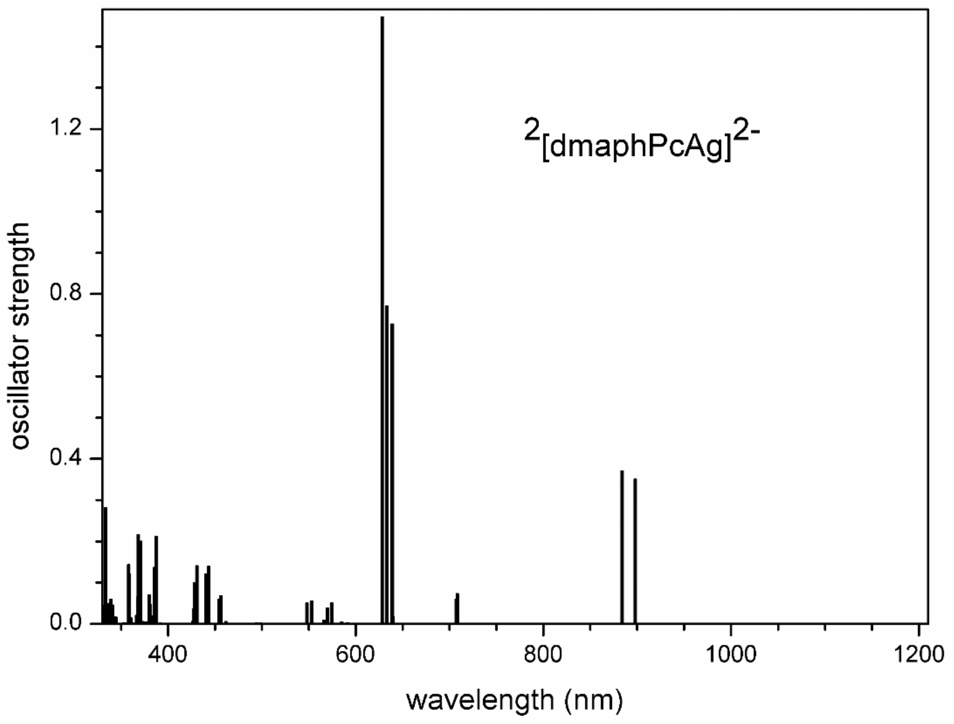

Figure S16. TD-DFT calculated electron transitions in ^{2}[dmaphPcAg]$^{2-}$ in $CHCl_3$.

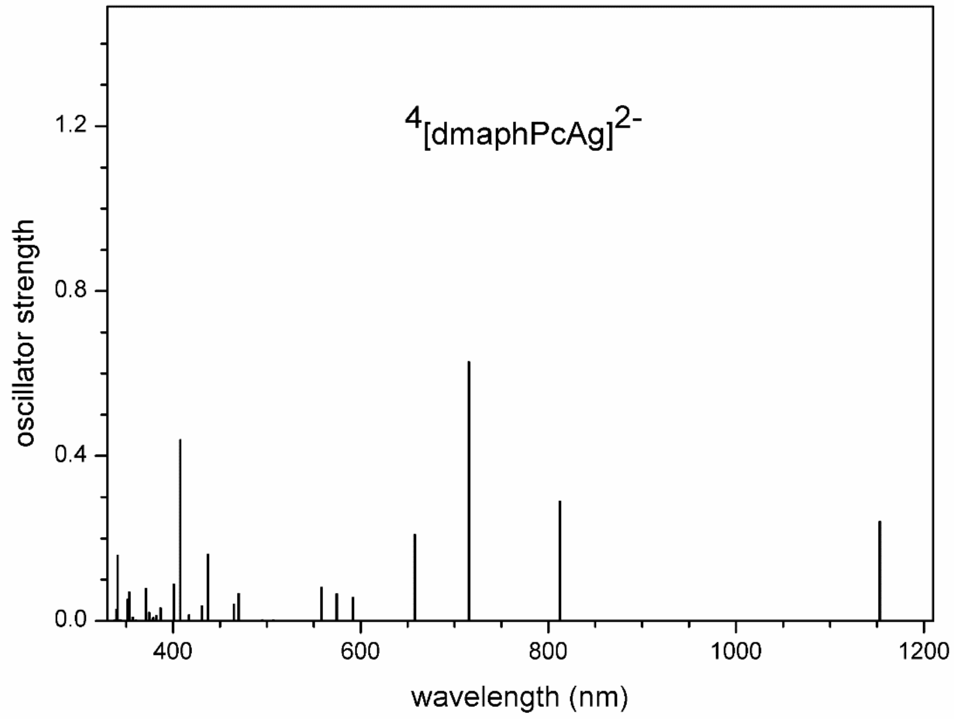

Figure S17. TD-DFT calculated electron transitions in ^{4}[dmaphPcAg]$^{2-}$ in $CHCl_3$.

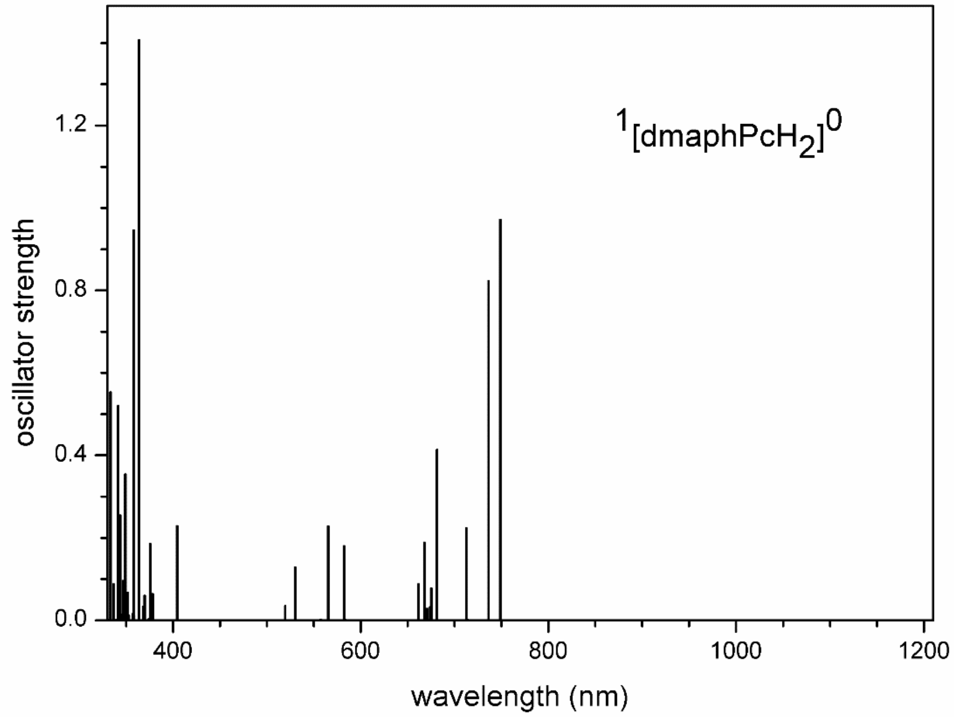

Figure S18. TD-DFT calculated electron transitions in ^{1}[dmaphPcH_2]^{0} in $CHCl_3$.

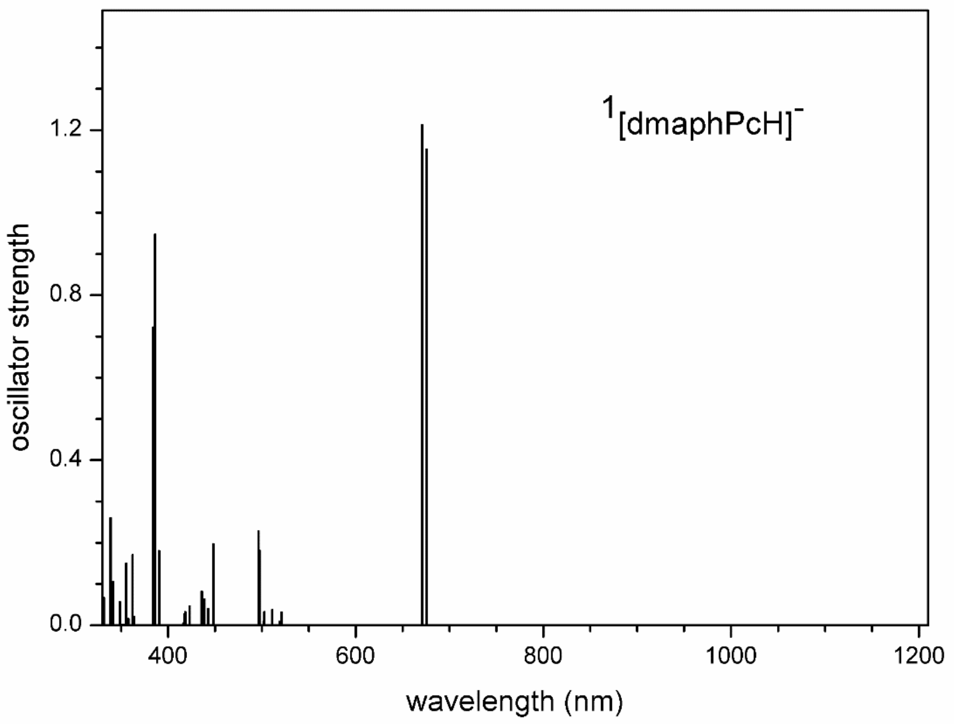

Figure S19. TD-DFT calculated electron transitions in ^{1}[dmaphPcH]$^-$ in $CHCl_3$.

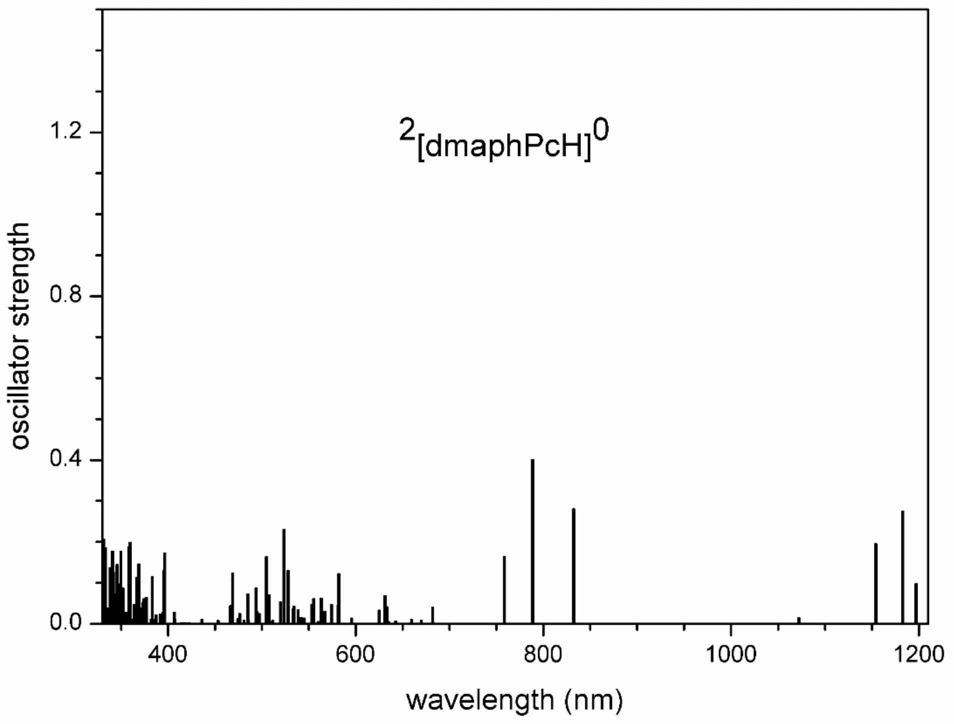

Figure S20. TD-DFT calculated electron transitions in ^{2}[dmaphPcH]^0 in $CHCl_3$.

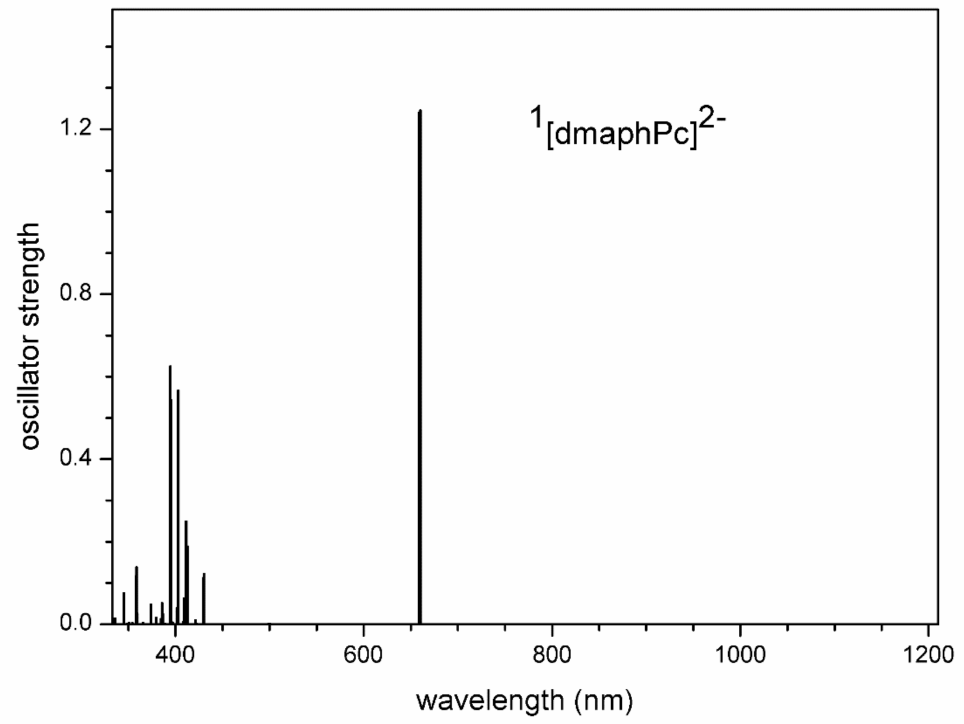

Figure S21. TD-DFT calculated electron transitions in ^{1}[dmaphPc]$^{2-}$ in $CHCl_3$.

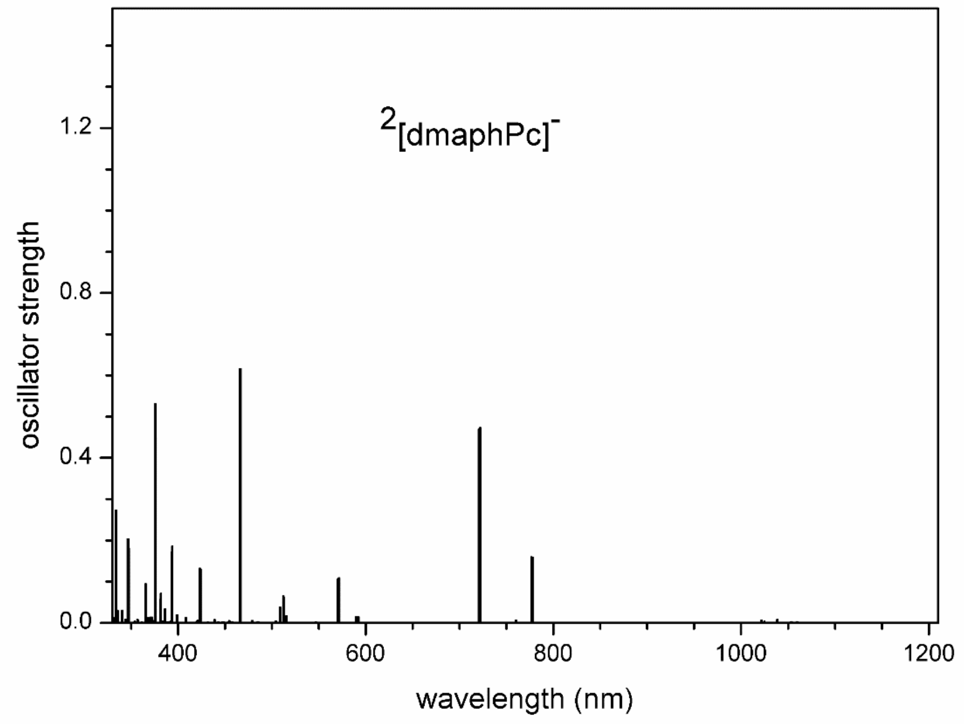

Figure S22. TD-DFT calculated electron transitions in ^{2}[dmaphPc]$^{-}$ in $CHCl_3$.

References

1. Breloy, L.; Alcay, Y.; Yilmaz, I.; Breza, M., Bourgon, J.; Brezová, V.; Yagci, Y., Versace, D.-L. Dimethyl amino phenyl substituted silver phthalocyanine as a UV- and visible-light absorbing photoinitiator: in situ preparation of silver/polymer nanocomposites. Polym. Chem. **2021**, 12, 1273-1285.
